# Occupational post-traumatic stress disorder: an updated systematic review

**DOI:** 10.1186/s12889-020-08903-2

**Published:** 2020-05-24

**Authors:** Wanhyung Lee, Yi-Ryoung Lee, Jin-Ha Yoon, Hye-Ji Lee, Mo-Yeol Kang

**Affiliations:** 1 Department of Occupational and Environmental Medicine, Gil Medical Center, Gachon University College of Medicine, Incheon, Republic of Korea; 2 Department of Occupational and Environmental Medicine, Seoul St. Mary’s Hospital, College of Medicine, The Catholic University of Korea, 222, Banpo-daero, Seocho-gu, Seoul, 06591 Republic of Korea; 3 The Institute for Occupational Health, Yonsei University College of Medicine, Seoul, Republic of Korea; 4 Department of Preventive Medicine, Yonsei University College of Medicine, Seoul, Republic of Korea; 5 Occupational Safety and Health Research Institute, Ulsan, Republic of Korea

**Keywords:** Stress disorders, post-traumatic, Workplace trauma, Risk factors, Literature review

## Abstract

**Background:**

Although numerous studies on occupational post-traumatic stress disorder (PTSD) have been conducted prior to the 1950–2010 seminal systematic review by Skogstad et al., the prevalence, risk factors, and impact of this disorder following traumatic events in occupational settings remain unclear. This study aims to address this knowledge gap by reviewing the literature published after 2010.

**Methods:**

We reviewed literature from databases such as PubMed and Google Scholar using PRISMA guidelines to identify studies that address occupational PTSD and examined the status (prevalence or incidence), the risk factors, and the health effects of PTSD among workers.

**Results:**

In total, 123 articles were identified, and finally, 31 (25.2%) articles were selected after excluding duplicates. Various occupational traumatic physical events were reported such as natural or manmade disaster, explosion, accident, handling refugee corpses, or bullying at work. Risk of PTSD was closely associated with working conditions, severity of injury, history of mental disorder, occurrence of psychiatric symptoms at the time of the event, personality, interpersonal relationships, etc. Workers with PTSD were likely to experience a deterioration of physical and psychological health and impairment of social and occupational functioning.

**Conclusions:**

Our review suggests that many workers remain highly vulnerable to occupational PTSD and its consequences.

## Background

Exposure to traumatic events in the workplace is common [1]. Although approximately 1.5% of workers reported being involved in a disastrous event or other accident at work, these events may be underreported and as a result, a large number of workers are exposed to accidents at work that may result in physical and psychological trauma [2]. However, there is substantial heterogeneity in the distribution of exposure to traumatic events in all types of occupations. Several studies reported that there are certain occupations in which a large number of workers are consistently exposed to large-scale traumatic events such as fatal accidents, mass disasters, the threat of death and injury, death of colleagues, witnessing death, suffering and injury, and assault [3].

Post-traumatic stress disorder (PTSD) is a mental health condition that is triggered by a terrifying event such as experiencing or witnessing a traumatic event involving actual or threatened death or serious injury. While there is growing concern that certain workers are at increased risk of PTSD, little is known about the nature and impact of PTSD on the mental health of the worker. Hence, further study is warranted to identify the impact of PTSD on physical health and determine various methods that can help reduce stress.

Although a large number of studies on occupational PTSD have been conducted, the prevalence, risk factors, and impact of this disorder in occupational settings remain unclear. Moreover, plenty of heterogeneity remains in the methodology of the studies. Hence, extreme caution is needed when drawing conclusions from these studies.

Skogstad et al. conducted an in-depth review of studies conducted on occupational groups that are at a high risk of developing work-related PTSD [3]. In this review, MEDLINE was searched for literature published in 1950–2010, while PsycINFO was searched for literature published in 1967–2010. In total, 140 eligible articles were selected. The results showed that work-related traumatic events are frequent in firefighters, ambulance personnel, police officers, healthcare professionals, train drivers, journalists, sailors, divers, and employees in banks, post offices, or stores. Moreover, mental health problems that occurred prior to the traumatic event and weak social support increased the risk of PTSD. From these findings, they suggested that prevention of work-related PTSD includes a sound organizational and psychosocial work environment, systematic training of employees, social support from colleagues and managers, and proper follow-up of employees after a critical event.

The number of studies on occupational PTSD, in addition to PTSD-related articles, has significantly increased since 2010. Therefore, we identified the need to update the literature review conducted since 2010. This update is of fundamental importance to research as it is aimed at advancing the knowledge on the prevention of occupational PTSD in the current industrial environment. This review was conducted based on previously published reviews related to the topic [3]. We aimed to update and expand on previous reviews as well as identify key challenges in the extant literature. Moreover, we aimed to comprehensively and critically assess previous studies on the prevalence, risk factors, and effect of PTSD following work-related traumatic events. We hope that this effort will help in improving our understanding of occupational PTSD and guide future research.

## Methods

### Search strategy

A literature search was conducted such as PubMed and Google scholar to determine all published reports on occupational PTSD according to the Preferred Reporting Items for Systematic reviews and Meta-Analyses guidelines [4]. We used the following search terms: (PTSD OR “post traumatic stress disorder” OR “posttraumatic stress disorder” OR “post-traumatic stress disorder” OR “acute stress disorder” OR “acute stress reaction”) AND (work* OR occupation* OR industry* OR employ*). A literature search was performed to identify relevant articles published in databases from August 1, 2010, to August 31, 2018. Previously, only 140 occupational PTSD-related articles published from January 1, 1950, to July 31, 2010, were reviewed [3].

### Inclusion and exclusion criteria

We included studies that satisfied the following criteria: studies (1) based on the working population, (2) with individuals whose PTSD was diagnosed by a medical professional, (3) categorized as original articles, and (4) that were published in English in a peer-reviewed journal. Exclusion criteria were as follows: articles (1) categorized as reviews, notes, commentaries, or editorials related to occupational PTSD; (2) based on retirees or adolescents; (3) in which PTSD was not the variable of interest; or (4) that described only the guidelines for clinical treatment or the study design.

### Selection and organization

**Fig. 1:**
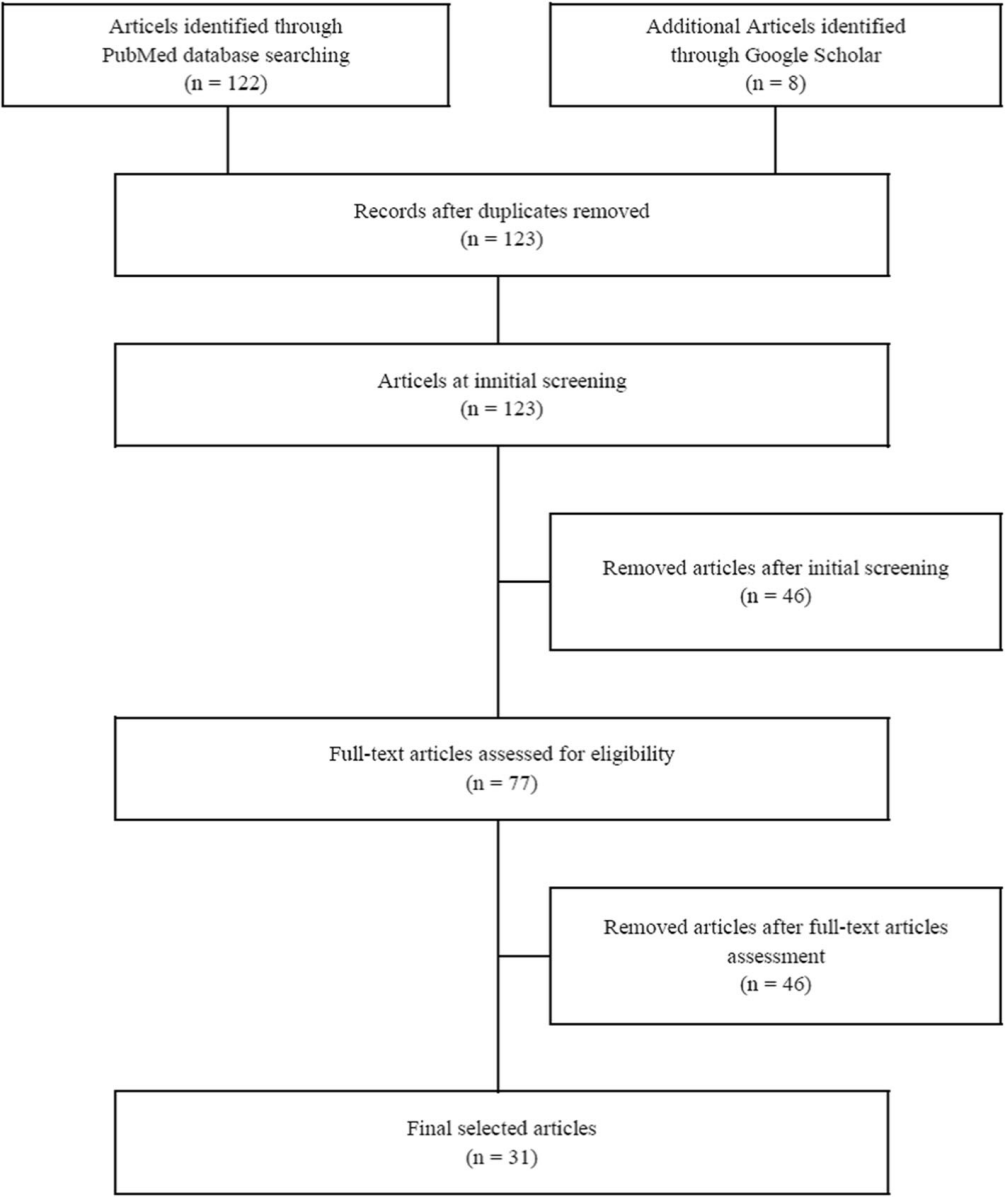
Flow diagram illustrating the article selection process based on the Preferred Reporting Items for Systematic reviews and Meta-Analyses guidelines

After searching the indicated databases as described above, the duplicate articles were removed. All articles were initially screened to select the relevant studies using the inclusion and exclusion criteria based on titles and abstracts. After the initial screening, the remaining articles were checked to determine eligibility. Consequently, quality assessments were conducted for the eligible studies (Supplementary Table). The full-text screening was performed by all authors to determine which of the articles fulfilled the inclusion criteria and achieved the purpose of the current study based on unanimous agreement. A flow chart of the process of article selection is displayed in Fig. 1.

We gathered the articles that addressed the following issues: (1) the status (prevalence or incidence) of PTSD among workers, (2) the risk factors of PTSD among workers, and (3) the effect PTSD on the health of the workers.

## Results

Overall, 123 PTSD-related articles that were published from August 1, 2010, to August 31, 2018, were found after excluding duplicates. About 46 articles were removed after further screening as the quality and scope of these studies did not fit those of the current study. Furthermore, 46 articles were excluded after full-text assessment as they were not original articles (*n* = 7), were not conducted in the working group (*n* = 16), did not involve a PTSD-focused investigation (*n* = 21), or were not within the scope of the current study (n = 2). Finally, 31 articles were selected.

These 31 studies were divided into 3 groups: 9 studies reported on the status of PTSD among workers, 14 reported on the risk factors of PTSD among workers, and 8 reported on the effect of PTSD on the health of the workers. We have described the studies included in detail.

### Status of PTSD among workers

Nine studies reported on the prevalence of occupational PTSD among workers on duty after a disaster, employees working in emergency services, and workers who experienced work-related trauma (Table 1). The prevalence of occupational PTSD varies greatly among studies (8.4–41.1%) due to variation in the definition of PTSD, the type of traumatic event, the period after exposure, and differences in occupation.

**Table 1:** Summary of published studies that fulfilled the inclusion criteria for the status of post-traumatic stress disorder among workers

First author.	Year	Country	Study design	Trauma	Participants	Trauma assessment	Prevalence or incidence
Sakuma, A	2015	Japan	Cross-sectional	Working after earthquake	1294 disaster relief and reconstruction workers (327 firefighters, 610 local municipality workers, and 357 hospital medical workers)	PCL-S ≥ 44, 14 months after the earthquake	Local municipality workers (9.0%) Medical workers (9.3%) Firefighters (2.6%)
Lee, JH	2016	Korea	Cross-sectional	Working related factors	3817 police officers with a traumatic event over a 1-year period	IES ≥26	All workers (41.1%) Inspector group (46.0%): assistant inspector group (42.7%) Intelligence and national security Division (43.6%), Police precinct (43.5%): traffic affairs Management department (43.3%)
Shi, L	2017	China	Cross-sectional	Physical violence	2706 healthcare workers from 39 public hospitals	PCL-C ≥ 50	All workers (28.0%)
Maslow, CB	2015	USA	Longitudinal	Working after 911	16,488 rescue, recovery, and clean-up workers	PCL-C	Low-stable group (53.3%) Moderate-stable group (28.7%), Moderate-increasing group (6.4%) High-decreasing group (7.7%) High-stable group (4.0%)
Carmassi, C	2016	Italy	Cross-sectional	Working related factors	110 emergency staff	Trauma and Loss Spectrum-Self Report	All (15.7%) Doctor (6.5%) Nurses (15.7%) Healthcare assistants (15.7%)
Fichera, GP	2015	Italy	Cross-sectional	Bank robbery	383 bank employee victims of robbery	IES > 34	13% at 45 days after the robbery
Cukor, J	2011	USA	Longitudinal	Working after 911	Clean-up and restore workers; 1) *N* = 727, 2) *N* = 2626	1) CAPS 2) PCL-C	1) 8.4% at 4-years after 911, 5.8% at 6 years after 911 2) 4.8% at 4-years after 911, 2.4% at 6 years after 911
Bromet, EJ	2015	USA	Longitudinal	Working after 911	3231 World Trade Center responders (957 non-traditional and 2274 police)	DSM-IV	9.7% current, 7.9% remitted, and 5.9% partial PTSD
Shamia, NA	2015	UK	Cross-sectional	War in Gaza	274 nurses	PCL-S:	19.7% at 2 years after the war

PCL-S: PTSD checklist-specific version

IES: Impact of Event Scale

PCL-C: PTSD Checklist-Civilian Version

CAPS: Clinician-Administered PTSD Scale

DSM-IV: Diagnostic and Statistical Manual of Mental Disorders 4th Edition

Workers on duty after a disaster had a high prevalence of PTSD. After the terror attacks on the World Trade Center on September 11, 2001, the prevalence of clinician-measured PTSD 4 years post-trauma was 8.4% [5]. After 11–13 years, 9.7% had current, 7.9% had remitted, and 5.9% had partial PTSD [6]; the definition of PTSD for this study was taken from The Diagnostic and Statistical Manual of Mental Disorders (DSM) 4th Edition. Previous study examined the prevalence of PTSD among 16,488 rescue and recovery workers 8–9 years after the 9/11 attack. Five groups had similar score trajectories in the PTSD Checklist (PCL): low stable (53.3%), moderate stable (28.7%), moderate increasing (6.4%), high decreasing (7.7%), and high stable (4.0%) [7]. Fourteen months after the Great East Japan Earthquake, the prevalence of PTSD (PCL-specific version score ≥ 44) was 9.0% among 610 municipalities, 9.3% among 357 medical workers, and 2.6% among 327 firefighters [8]. The prevalence of PTSD after 2 years was 19.7% among 274 nurses who worked in war zones in Gaza [9].

The prevalence of PTSD was 41.1% (Impact of Event Scale revised Korean version; IES-K score ≥ 26) among 3817 police officers in Korea who experienced traumatic events over a 1-year period [10], 28.0% (PCL-Civilian Version score ≥ 50) among 2706 healthcare workers from Chinese hospitals who experienced physical violence [11], 15.7% (DSM-V PTSD diagnosis using Trauma and Loss Spectrum Self-Report; TALS-SR) among the 83 emergency staff in an Italian hospital [12], and 13% (IES score > 34) among 383 bank employees who were victims of robbery in Italy [13].

### Risk factors of PTSD among workers

Occupational PTSD can be associated with negative working conditions such as long working hours, layoffs, workplace stress, the severity of the injury, frequency of exposure, marital status, history of mental disorder or occurrence of psychiatric symptoms at the time of the event, personality, negative interpersonal relationship, etc.

Several previous studies suggested the risk factors of PTSD for workers experiencing industrial accidents (Table 2). Among injured workers who survived after a major factory collapse in Bangladesh, those employees who worked 70 h a week (adjusted odds ratio [aOR] = 2.4; 1.1–5.3), had a concussion injury (aOR = 3.7; 1.4–9.8), and were married (aOR = 3.2; 1.3–8.0) [14] had an increased risk of developing PTSD. After an industrial explosion, the risk factors of PTSD among the nearby workers included trauma, history of mental disorder or occurrence of psychiatric symptoms at the time of the event [15, 16], proximity to explosion site, non-managerial occupation, age over 50 years in both sex, layoffs (aOR = 2.6; 1.5–4.5), and unusable workplace after the explosion (aOR = 1.8; 1.1–2.8) in men [17]. A Canadian study evaluating the health of urban public transit employees after experiencing traumatic events at the workplace indicated difference in PTSD severity according to the severity of depression, gender, ethnicity, and workplace stress [18].

**Table 2:** Summary of published studies that fulfilled the inclusion criteria for risk factors of post-traumatic stress disorder among workers

First author.	Year	Country	Study design	Trauma	Participants	Trauma assessment	Estimate of risk
Fitch, T	2015	Bangladesh	Cross-sectional	Factory building collapse	181 survivors at 1 year post factory collapse	PCL-S ≥ 50	(OR, 95% CI) Married (3.2, 1.3–8.0), More than 70 working hours/week (2.4, 1.1–5.3), Higher job positions (2.6, 1.2–5.6), Concussion injury (3.7, 1.4–9.8)
James, L	2018	USA	Cross-sectional	Chronic exposure to critical incidents in workplace	355 prison workers	PCL for DSM-5	(Regression coefficients β, p-value) 1) Risk factor Being seriously injured (3.13, < 0.01), Encountering an inmate recently sexually assaulted (1.29, < 0.01), Being often placed in unnecessary danger (1.79, < 0.01), Being often unclear about what is expected of them (1.05, < 0.01). 2) Protective factor Being happy with job assignments (−1.49, < 0.01), Having positive relationships with supervisors (− 1.39, < 0.01), Having positive relationships with co-workers (− 1.46, < 0.01).
Shah, R	2017	Canada	Longitudinal	Workplace traumatic event	141 urban public transit employees	SCID-I	Factors which were significantly associated with PTSD severity (Regression coefficients β, *p*-value) Severity of depression (0.66, 0.01), Female (3.31, 0.02), Ethnicity (13.33, 0.01), Workplace related stress (− 0.30, 0.02).
Geronazzo-Alman, L	2017	USA	Cross-sectional	Cumulative exposure to work-related traumatic events	209 first responders	PCL-C	(Adjusted OR, 95% CI) Frequency of exposure (2.0, 1.2–3.3), Variety of exposure (2.8, 1.5–5.5), Nomothetic severity of exposure (2.9, 1.5–5.7), Idiographic severity of exposure (5.2, 2.4–11.3).
Schenk, EJ	2017	China	Cross-sectional	Working after earthquake	337 medical rescue workers who performed within the first 3 months of the event	IES-R ≥ 33	(Adjusted OR, 95% CI) Injured during rescue work (2.7, 1.4–5.1), Experienced a water shortage (3.0, 1.4–6.6), Disconnected from family or friends during rescue work (1.7, 0.8–3.7).
Bogaerts, S	2013	Netherlands	Cross-sectional	Intracolleague aggression	174 prison workers	The Self-Rating Inventory for PTSD	Degree of type D personality (F = 21.9, *p* < 0.01)
Spence Laschinger, HK	2015	Canada	Cross-sectional	Workplace bullying	874 nurses (244 new graduate nurses and 630 experienced nurses)	Primary care PTSD screen	(Regression coefficients β, p-value) 1) Risk factor: workplace bullying among new graduate nurses (0.51, < 0.01), among experienced nurses (0.52, < 0.01) 2) Protective factor: psychological capital among new graduate nurses (−0.25, < 0.01) among experienced nurses (− 0.20, < 0.01)
Taymur, I	2014	Turkey	Longitudinal	Industrial Explosion	157 workers nearby the explosion building	CAPS	Factors showing significant differences using Pearson’ chi-squared test 1) After 1 month History of psychiatric disorder, physical injury, acquaintances among the dead/injured, being involved in the incident, and having seen dead people 2) After 6 months: physical injury, acquaintances among the dead/ injured, being involved in the incident
Chatzea, VE	2018	Greece	Cross-sectional	Working during the European refugee crisis	217 rescue workers	PCL-C ≥ 50	(Adjusted OR, 95% CI) Female (2.1, 1.0–3.3), Single/divorced/widower (3.4, 2.2–4.6), Age (1.9, 1.8–2.1), Operation periods (2.1, 1.9–2.3), Duration of shifts (3.1, 2.5–3.7), Handling dead adults (2.8, 2.6–3.0), Handing dead children (2.9, 2.8–3.0).
Diene, E	2012	France	Cross-sectional	Industrial factory explosion	13,129 economically active persons in the immediate and peripheral area of industrial disaster	IES-R ≥ 33	(Adjusted OR, 95% CI) 1) Men Employees (4.3, 2.3–7.8), Factory workers/laborers (3.7, 1.8–7.6), Temporary layoff (2.6, 1.5–4.5), Unusable workplace (1.8, 1.1–2.8), Attendance at emergency department (4.1; 2.8–6.1), < 1.7 km to explosion site (3.6, 1.6–8.1), ≥50 years old (2.8, 1.3–5.9) 2) Women Artisan (2.7, 1.3–5.7), Employees/factory workers/laborers (2.2, 1.4–3.5), Attendance at emergency department (3.0, 2.2–4.4), Reporting of an occupational accident (1.5, 1.1–2.2), < 1.7 km to explosion site (3.0, 1.2–7.3), ≥50 years old (1.9, 1.1–3.1)
Sifaki-Pistolla, D.	2017	Greece	Cross-sectional	Working during the European refugee crisis	217 rescue workers	PCL-C ≥ 50	(Adjusted OR, 95% CI) Female (2.2, 1.1–3.4), Single/divorced/widower (3.5, 2.3–4.7), > 40 years old (3.8, 2.5–5.1), > 14 operation days (2.3, 1.4–3.2), > 4 shift hours/day (3.9, 3.1–4.7), Handling over 6 dead refugees (3.4, 2.3–4.5), Handling dead children (3.2, 1.9–4.4).
Song, J. Y.	2018	Korea	Cross-sectional	Chemical release disasters	237 workers in industrial complex	IES-R ≥ 24	(Adjusted OR, 95% CI) Alcohol dependence (3.1, 1.3–7.6), Psychiatric symptom at the time of the accident (5.3, 1.8–15.6) Workers with high perceived stress scale scores (8.7, 2.3–33.2)
Noda, Y.	2018	Japan	Cross-sectional	Working after earthquake	220 rescue workers	IES-R ≥ 24	(Coefficients β, p-value) 1) Higher level of education Intrusion (−0.17, 0.02) Avoidance (− 0.18, 0.03) Hyperarousal (− 0.18, 0.02) 2) Resilience Intrusion (− 0.18, 0.02) Avoidance (− 0.16, 0.02) Hyperarousal (− 0.26, < 0.01)
McCanlies, EC.	2014	USA	Cross-sectional	Working after hurricane	114 police officers	PCL-C	(Coefficients β, *p*-value) 1) Resilience (− 0.65, < 0.01) 2) Satisfaction with life (− 0.55, < 0.01) 3) Gratitude (− 0.67, < 0.01) 4) Post-traumatic growth (0.09, 0.55)

PCL-S: PTSD Checklist-Specific Version

SCID-I: Structured Clinical Interview for the DSM-IV Axis I Disorder

IES-R: Impact of Event Scale-Revised

PCL-C: PTSD Checklist-Civilian Version

OR: Odds ratio

95% CI: 95% confidence interval

The risk factors of PTSD among workers on duty after earthquake were as follows: injury, experience with water shortage, disconnection from family and friends during the response, having passive coping styles, exhibiting neurotic personalities in case of Chinese medical rescue workers [19], lower education status, and resilience in case of Japanese rescue workers [20]. The risk factors of PTSD among workers on duty during the European refugee crisis were as follows: marital status (single/divorced/widower), age, long operation period (> 14 days, aOR = 2.3;1.4–3.2), long shift hours (> 4 h/day, aOR = 3.9; 3.1–4.7), and handling dead refugees (> 6 refugees, aOR = 3.4; 2.3–4.5) and dead children (> 1 children, aOR = 3.2; 1.9–4.4) [21, 22]. One study conducted on first responders suggested that the variety of exposure and the frequency of exposure were associated with PTSD [23]. The police officers who worked after Hurricane Katrina showed significantly decreased risk of PTSD, increased level of resilience, satisfaction with life, and gratitude [24].

Prison workers, who were exposed to critical incidents, had ambiguity in their job role, had negative relationships with supervisors and co-workers, and had a type D (distressed) personality [25, 26], were at an increased risk of developing PTSD. Workplace bullying was a significant risk factor for PTSD among hospital nurses [27].

### Effect of PTSD on the health of workers

Workers with PTSD are more likely to experience worsening of health status, problems in social and occupational functioning, early retirement, and job loss than those with no PTSD (Table 3). Among rescue and recovery workers with asthma, PTSD comorbidity showed worse asthma control, higher rates of inpatient healthcare utilization, and poorer quality of life than those with sub-threshold or no PTSD [28]. In a cohort of workers exposed to the 9/11 attack, PTSD was significantly associated with bronchodilator response at baseline (aOR = 1.43; 1.19–1.72) and predicted incident asthma (aOR = 2.41; 1.85–3.13) [7].

**Table 3:** Summary of published studies that fulfilled the inclusion criteria for the effect on workers with post-traumatic stress disorder

First author.	Year	Country	Study design	Trauma	Participants	Effect	Results
Giosan, C	2015	USA	Cross-sectional	Working after the 9/11 attack	2453 utility workers	CAPS	PTSD severity was significantly associated with sleep disturbance (β = 0.52, p-value < 0.01).
Mindlis, I	2017	USA	Longitudinal	Working after 911	181 rescue and recovery workers with asthma	Asthma morbidity	PTSD patients showed - Worse asthma control (mean difference = 0.57, 95% CI: 0.12–1.02) - Poorer asthma quality of life (mean difference = − 0.83, 95% CI: −1.32–0.34) - Higher rates of inpatient healthcare utilization due to asthma (adjusted OR = 11.9, 95% CI: 3.5–40.1).
Hunnicutt-Ferguson, K	2018	USA	Longitudinal	Working after the 9/11 attack	Clean-up and restore workers who met the criteria for PTSD or subthreshold PTSD at baseline (*N* = 514), 1-year (*N* = 289), and 2-year follow-up (*N* = 179)	Functional impairment and subjective distress	PTSD severity was positively associated with - Subjective distress, - Social impairment - Occupational impairment
Yu, S	2016	USA	Longitudinal	Working after the 9/11 attack	7662 rescue and recovery workers	Early retirement and job loss	(Adjusted OR, 95% CI) Chronic conditions and PTSD comorbidity increased OR for 1) Early retirement - Three chronic conditionswithout PTSD (1.3, 0.6–2.7) - Three chronic conditions with PTSD (2.1, 1.2–3.9) 2) Job loss - Three chronic conditionswithout PTSD (3.2, 1.6–6.5) - Three chronic conditions with PTSD (10.7, 6.7–17.2)
de la Hoz, RE	2016	USA	Longitudinal	Working after the 9/11 attack	11,481 workers and volunteers who performed rescue, recovery, and service restoration duties	BDR, incident asthma	(Adjusted OR, 95% CI) Mean f/u period 4.95 years. 1) PTSD - > BDR - At baseline, all participants (*N* = 11,481): (1.4, 1.2–1.7) 2) PTSD - > incident asthma - F/u visits, never smokers without asthma at baseline (*N* = 3757): (2.4, 1.9–3.1).
Luft, BJ	2012	USA	Longitudinal	Working after 911	8508 police and 12,333 non-traditional responders	Respiratory symptoms, and pulmonary function test	1) Correlation: Lower respiratory symptoms - Police (r = 0.28) - Non-traditional responders (r = 0.27)2) No correlation: lung function - Police (r = 0.03) - Non-traditional responders (r = 0.03)
Kerai, S	2017	Pakistan	Cross-sectional	Working related factors	507 emergency medical service personnel (doctors 37, nurses 202, drivers 211, paramedics 57)	Work performance: number of late arrivals to work, number of days absent, number of days sick, adherence to protocol, and patient satisfaction over a period of 3 months	No statistically significant association was found between PTSD and work performance in multiple logistic regression.
Kotov, R	2015	USA	Longitudinal	Working after 911	18,896 responders (8466 police and 10,430 non-traditional)	Respiratory symptoms	Lower respiratory symptoms was positively correlated with - Worked in dust cloud, - Long hours on site

CAPS: Clinician-Administered PTSD Scale

BDR: Bronchodilator response

OR: Odds ratio

95% CI: 95% confidence interval

Among male utility workers on duty after the 9/11 attack, PTSD was significantly associated with sleep disturbance (β = 0.52, *p*-value < 0.01) [29]. Among rescue and recovery workers on duty after the 9/11 attack, workers with PTSD were more likely to experience early retirement and job loss [30] than those with no PTSD, and PTSD severity was positively associated with subjective distress and deficits in social and occupational functioning, over time [31]. Among policemen who responded to the 9/11 attacks and non-traditional responders, a significant relationship was observed between PTSD and lower respiratory symptoms [32, 33]. Meanwhile, no significant association was found between PTSD and work performance among emergency medical service personnel in Pakistan [34].

## Discussion

This updated review of the existing research literature on occupational PTSD shows that PTSD prevalence varies among different occupations according to traumatic events. Various types of occupational traumatic physical events have been reported: natural or manmade disaster, explosion, accident, and handling dead refugees. Moreover, psychological traumas including bullying became an important risk factor of PTSD. The risk of PTSD was closely associated with the severity of the injury, working conditions, marital status, history of mental disorder or occurrence of psychiatric symptoms at the time of the event, personality, interpersonal relationship, etc. Workers with PTSD are likely to experience worsening of physical and psychological health as well as deficits in social and occupational functioning, early retirement, and job loss.

It is difficult to assess the full extent of the occurrence of traumatic events in the workplace due to the lack of reliable data. However, several incidents that occur at work meet the criteria for traumatic events. Therefore, several workers seemed to be exposed to traumatic events in the workplace, which increases the risk of developing PTSD or other trauma-related disorders.

Although it is commonly acknowledged that staff working in the emergency services face traumatic and distressing situations as a part of their normal working life, they still have an increased risk of developing mental health problems [5, 7, 12, 19–22, 28, 31, 34, 35]. However, little is known about the psychological problems that workers, who experience other forms of traumatic events in their workplace, may develop.

In addition, PTSD is more likely to occur in police officers exposed to traumatic events as part of their work, especially after natural disasters [24, 36]. Among police officers, the rate of PTSD was estimated at 43.6% [10]. It has been found that police officers’ self-resilience can affect the severity and intensity of PTSD symptoms and is a protective factor against the development of post-traumatic stress disorder symptoms [37].

Once workers recognize it, their reactions to traumatic events fall into three main phases: a) immediate reactions at the time of the trauma, b) acute reactions a month after the trauma, and c) chronic or long-term reactions. Each phase involves a number of characteristic responses. One of the major findings of trauma from previous research is that the majority of people are able to deal with their traumatic experiences well and rarely develop PTSD after a traumatic experience [2]. These findings suggest that for some workers, PTSD is an outcome of failure in recovery rather than the natural results of the traumatic exposure [37, 38]. Studies have suggested that many factors determine the magnitude and duration of trauma responses. Main factors include the intensity and nature of the traumatic event, the perception of the trauma by the worker, the level of training and preparation to meet the demands of the trauma, and the availability of appropriate support [14–17, 19, 20, 22, 23, 25–27, 29].

When chronic repeated exposure to traumatic events of lower magnitude in the daily occupational routine occurs over a period of time, the risk of accumulating risk also has a devastating impact on workers’ mental health similar to that on workers who have been exposed to single catastrophic event. A key concept in the cumulative risk of repeated exposure is fear conditioning [39]. This is the process by which an individual’s responsiveness to trauma-related signals gradually increases. In the aftermath of traumatic exposure, there are critical periods in which irreversible neuronal changes can occur in people with PTSD. Many studies highlight these cumulative risks, representing major challenges in the work environment, with the goal of minimizing this process in the work environment [23, 25, 27].

In many workplaces, it would be advantageous if managers could notice signs that a worker is experiencing typical PTSD reactions. PTSD patients may use alcohol, drugs, caffeine, or nicotine or experience social withdrawal, depression, somatic distress, performance deterioration, interpersonal, and/or family conflict, etc. [1, 7, 28, 30, 31, 34].

### Methodological considerations

Most studies on work-related PTSD were primarily based on survey data and have a cross-sectional study design, which precludes inferences of causal explanations. Longitudinal studies are warranted to confirm the causal nature of the relationship. Meanwhile, errors can occur in retrospective studies due to selective bias. Particularly, having a negative mood strongly disturbed the retrospective self-reported recollection of the life of a worker (ref: selective bias in retrospective self-reported of negative mood). Disease status can affect the recollection of memories by an individual as unhealthy people tend to underestimate the quality of their past life (ref: Breast cancer survivors’ recollection of their quality of life: identifying determinants of recall bias in a longitudinal population-based trial).

Another methodological issue is the definition of the traumatic event and the timing of the PTSD assessment in relation to this event, which makes it difficult to draw a definitive conclusion from the findings of various studies. In most of the studies included in our review, which evaluated the prevalence of occupational PTSD, assessment of PTSD symptoms was completed using constructed questionnaires such as Clinician-Administered PTSD Scale, Impact of Event Scale (IES), or PCL with cut-off values indicating a diagnosis of PTSD. However, a strict application of the diagnostic criteria is essential in research on PTSD. There could be confusion between psychopathology and normal reactions to psychosocial stress or other psychiatric problems when applying checklists for subjective symptoms. Participants whose symptom scores are above these thresholds for caseness cannot be successfully diagnosed with PTSD. Therefore, there is a possibility of overdiagnosis of PTSD when the diagnosis is based on the symptom checklist rather than the criteria described in DSM-5 or diagnosis by a physician. Although a symptom checklist is a simple tool for assessing PTSD, it cannot be used as a substitute for full clinical diagnostic criteria [40]. Moreover, there is a risk of self-report bias in these studies, which could lead to an “artifactual covariance between the predictor and criterion variable” since the same person is assessing both measures [41]. In addition, a variety of different questionnaires have been applied across researches making a comparison of the results difficult.

The timing of PTSD assessment varied widely across the studies, although it is critical in understanding the reported prevalence of PTSD. According to DSM-5 criteria, it is necessary to stipulate both the onset of the PTSD symptoms and their duration. Acute PTSD can be defined after a symptom duration of at least 1 month after the traumatic event; if symptom duration exceeds more than 3 months, it is referred to as chronic PTSD. Meanwhile, if the PTSD symptoms occur at least 6 months after the traumatic event, it is referred to as delayed-onset PTSD. However, the timing of the traumatic event remains unclear and depends on an individual experiencing workplace trauma “to remember” when the PTSD symptoms first occurred.

## Conclusions

Occupational groups such as healthcare workers, police officers, prison workers, and emergency personnel are at increased risk of experiencing traumatic events that make them likely to develop PTSD. This condition can cause deterioration of physical and psychological health and lead to deficits in social and occupational functioning, early retirement, job loss, and in extreme cases, suicide. Certain factors such as personality traits, earlier psychiatric morbidity, poor working condition, and lack of social support, increase the risk for PTSD. PTSD may develop because of risk factors which may be common to other comorbidities. Therefore, these vulnerability factors should be assessed for the prevention of occupational PTSD. In this respect, management of the interventional factors from traumatic exposure in the workplace that contribute to the development of PTSD is particular challenging. Ideally, an occupational health service should provide readily accessible evidence-based treatment in a timely manner to those workers identified to be symptomatic as well as identify and manage the risks at an organizational level [3]. A decent work environment, social support from colleagues and managers, and proper follow-up of victims would be essential to prevent the development of PTSD from work-related trauma. However, only a few studies have examined the effectiveness of evidence-based treatments in different occupational settings.

Our review revealed that a number of groups were still highly vulnerable to PTSD, but it did not include a detailed explanation of the psychosocial, cultural, and economic factors that make an individual vulnerable to traumatic events. This review of the literature suggests that several areas need to be explored. An important issue for upcoming research would be to develop proper interventions against workplace trauma as well as treatment procedures, which can be used to limit the potential traumatic consequences of work-related traumatic events. Hence, future studies are warranted to provide better guidance on workplace trauma-specific interventions or therapeutic treatments. Future development of interventions would benefit from studies that clearly and explicitly identify populations at risk.

## Supplementary information

**Additional file 1: Table S1.** Newcastle-Ottawa Quality Assessment Scale of studies included in this review.

## Data Availability

Not applicable.

## Abbreviations

PTSD: Post-traumatic stress disorder
PCL: PTSD Checklist
IES: Impact of event scale
DSM: Diagnostic and statistical manual of mental disorders
aOR: Adjusted odds ratio
