# Supplementary material for: Occupational post-traumatic stress disorder: an updated systematic review
Source: BMC Public Health. 2020 May 24;20:768. doi: 10.1186/s12889-020-08903-2 (PMC7245752; doi:10.1186/s12889-020-08903-2)
Supplement: Supplementary file 1 — Additional file 1: Table S1. Newcastle-Ottawa Quality Assessment Scale of studies included in this review. [file 12889_2020_8903_MOESM1_ESM.docx]

**Supplementary Table.** Newcastle-Ottawa Quality Assessment Scale of studies included in this review

| First author. | Year | S1 | S2 | S3 | S4 | C | O1 | O2 | O3 | Total score |
| --- | --- | --- | --- | --- | --- | --- | --- | --- | --- | --- |
| Cross-sectional studies* | |  |  |  |  |  |  |  |  |  |
| Diene, E | 2012 | 1 | 1 | 0 | 1 | 2 | 1 | 1 | - | 7 |
| Bogaerts, S | 2013 | 1 | 0 | 0 | 1 | 2 | 1 | 1 | - | 6 |
| McCanlies, EC. | 2014 | 1 | 0 | 0 | 2 | 2 | 1 | 1 | - | 7 |
| Sakuma, A | 2015 | 1 | 1 | 0 | 1 | 2 | 1 | 1 | - | 7 |
| Fichera, GP | 2015 | 1 | 1 | 0 | 1 | 2 | 1 | 1 | - | 7 |
| Fitch, T | 2015 | 1 | 0 | 0 | 1 | 2 | 1 | 1 | - | 6 |
| Spence Laschinger, HK | 2015 | 1 | 0 | 0 | 2 | 2 | 1 | 1 | - | 7 |
| Giosan, C | 2015 | 1 | 1 | 0 | 2 | 2 | 1 | 0 | - | 7 |
| Shamia, NA | 2015 | 1 | 0 | 0 | 1 | 2 | 1 | 1 | - | 6 |
| Carmassi, C | 2016 | 0 | 0 | 1 | 1 | 1 | 1 | 1 | - | 5 |
| Shi, L | 2017 | 1 | 1 | 0 | 1 | 1 | 1 | 1 | - | 6 |
| Geronazzo-Alman, L | 2017 | 1 | 0 | 0 | 2 | 2 | 1 | 1 | - | 7 |
| Schenk, EJ | 2017 | 1 | 0 | 0 | 1 | 2 | 1 | 0 | - | 5 |
| Sifaki-Pistolla, D. | 2017 | 1 | 0 | 0 | 1 | 2 | 1 | 1 | - | 6 |
| Kerai, S | 2017 | 1 | 0 | 0 | 1 | 2 | 2 | 1 | - | 7 |
| James, L | 2018 | 1 | 0 | 0 | 1 | 1 | 1 | 1 | - | 5 |
| Chatzea, VE | 2018 | 1 | 0 | 0 | 2 | 2 | 1 | 1 | - | 7 |
| Song, J. Y. | 2018 | 1 | 0 | 0 | 1 | 2 | 1 | 1 | - | 6 |
| Noda, Y. | 2018 | 1 | 0 | 0 | 2 | 1 | 1 | 1 | - | 6 |
| Longitudinal studies | |  |  |  |  |  |  |  |  |  |
| Cukor, J | 2011 | 1 | 1 | 0 | 0 | 2 | 1 | 1 | 1 | 7 |
| Luft, BJ | 2012 | 1 | 1 | 0 | 0 | 2 | 1 | 1 | 1 | 7 |
| Taymur, I | 2014 | 1 | 1 | 0 | 0 | 1 | 1 | 0 | 1 | 5 |
| Maslow, CB | 2015 | 1 | 1 | 0 | 0 | 2 | 0 | 1 | 1 | 6 |
| Bromet, EJ | 2015 | 1 | 1 | 0 | 0 | 2 | 1 | 1 | 1 | 7 |
| Kotov, R | 2015 | 1 | 1 | 0 | 0 | 2 | 1 | 1 | 1 | 7 |
| Yu, S | 2016 | 1 | 1 | 0 | 1 | 2 | 0 | 1 | 1 | 7 |
| de la Hoz, RE | 2016 | 1 | 1 | 0 | 1 | 2 | 1 | 1 | 1 | 8 |
| Shah, R | 2017 | 0 | 1 | 1 | 0 | 2 | 0 | 1 | 1 | 6 |
| Mindlis, I | 2017 | 0 | 1 | 1 | 0 | 2 | 0 | 1 | 1 | 6 |
| Hunnicutt-Ferguson, K | 2018 | 1 | 1 | 1 | 0 | 2 | 0 | 1 | 0 | 6 |

S: selection, C: comparability, O: outcome

* Modified version of the Newcastle-Ottawa scale was used in the cross-sectional studies
